# Features extraction based on Naive Bayes algorithm and TF-IDF for news classification

**DOI:** 10.1371/journal.pone.0327347

**Published:** 2025-07-30

**Authors:** Li Zhang

**Affiliations:** School of Artificial Intelligence, Zhejiang College of Security Technology, Wenzhou, Zhejiang, China; Philadelphia University, JORDAN

## Abstract

The rapid proliferation of online news demands robust automated classification systems to enhance information organization and personalized recommendation. Although traditional methods like TF-IDF with Naive Bayes provide foundational solutions, their limitations in capturing semantic nuances and handling real-time demands hinder practical applications. This study proposes a hybrid news classification framework that integrates classical machine learning with modern advances in NLP to address these challenges. Our methodology introduces three key innovations: (1) Domain-Specific Feature Engineering, combining tailored n-grams and entity-aware TF-IDF weighting to amplify discriminative terms; (2) BERT-Guided Feature Selection, leveraging distilled BERT to identify contextually important words and resolve rare-term ambiguities; and (3) Computationally Efficient Deployment, achieving 95.2% of the accuracy of BERT at 1/52.4th of the inference cost. Evaluated on a balanced corpus of Sina News articles in 11 categories, the system demonstrates a test precision of 95.12% (vs. 84.43% for SVM+TF-IDF baseline), with statistically significant improvements confirmed by 5-fold cross-validation(*p* < 0.01). The critical findings reveal strong performance in distinguishing semantically distinct categories, while exposing challenges in fine-grained differentiation. The efficiency of the framework (2.1 inference latency) and scalability (linear utilization of CPU resources) validate its practicality for real-world deployment. This work bridges the gap between traditional feature engineering and transformer-based models, offering a cost-effective solution for news platforms. Future research will explore hierarchical classification and the adaptation of dynamic topics to further refine semantic boundaries.

## Introduction

### Research story

News classification is the process by which a computer maps the text with information into predetermined categories. As a core technology of text mining, it enables the accurate and rapid determination of the classification to which textual information belongs, addressing key tasks in Natural Language Processing (NLP). News classification is widely applied in various domains, including sentiment analysis, conversational behavior classification, spam detection, and personalized news recommendation systems [1]. Over the years, researchers have proposed numerous classification algorithms, ranging from traditional machine learning methods such as decision trees [2], support vector machines (SVM) [3], and Naive Bayes [4], to more advanced deep learning-based approaches. In recent years, deep learning models, including Convolutional Neural Networks (CNN) [5], Long-Short-Term Memory (LSTM) [6], and Recurrent Neural Networks (RNN) [7], have demonstrated superior performance in text categorization tasks. These models not only excel in text filtering but also achieve high accuracy in semantic analysis by capturing complex linguistic patterns. With the advent of transformer-based architectures, such as BERT (Bidirectional Encoder Representations from Transformers) [8] and GPT (Generative Pre-trained Transformer) [9], text classification has reached new heights. These models leverage pre-trained language representations and fine-tuning techniques to achieve state-of-the-art results across various NLP tasks, including news classification. Furthermore, the integration of knowledge graphs [10] and attention mechanisms [11] has further enhanced the ability of classification systems to understand context and relationships within text data.

### Problem statement

The application of NLP technology in journalism has revolutionized the way news is categorized and consumed. For example, platforms such as Google News utilize knowledge graphs combined with clustering algorithms to establish semantic connections between news articles, enabling personalized news recommendations [4]. Similarly, Netease News employs advanced algorithms, such as the the “Beyond Algorithm,” to accurately categorize news content based on user preferences and contextinformation [5]. Despite these advancements, most text classification algorithms still treat text labels as independent entities, often ignoring the rich contextual information embedded within the text [17]. This limitation hinders the ability of these systems to fully capture the nuances of news content, leading to suboptimal classification performance [19]. Furthermore, the rapid growth of online news data, coupled with the increasing demand for real-time classification, poses significant challenges for traditional methods [18].

To address these challenges, there is a pressing need to explore innovative approaches that leverage the latest advancements in NLP, such as transformer-based models, attention mechanisms, and multi-modal learning [20]. By effectively utilizing label information and incorporating contextual understanding, we can develop more robust and accurate news classification systems that cater to the dynamic needs of modern journalism.

Our hybrid framework distinguishes itself from previous work [21,25] through three key innovations:

1. Entity-aware TF-IDF weighting (α=0.3 in Eq. 4), which amplifies the named entities detected by NER.
2. Dynamic BERT attention masking (threshold >0.7) for rare-term disambiguation.
3. Parallel feature fusion architecture (section 3.3) that reduces latency by 40% compared to sequential pipelines.

This contrasts with the static weighting of [21] and the post hoc fusion approach of [25].

### Related works

Text classification remains a fundamental challenge in natural language processing, particularly for dynamic domains like news categorization where both efficiency and semantic understanding are crucial. While traditional approaches combining TF-IDF feature extraction with Naive Bayes classification provide a strong baseline [8,12], modern news analysis demands more sophisticated solutions that balance computational efficiency with state-of-the-art accuracy. In this work, we present an enhanced Sina news classification system that innovatively bridges classical machine learning with contemporary NLP advances through three key contributions:

#### Hybrid feature engineering framework.

We develop a novel feature space combining (a) domain-specific n-grams with (b) entity-enhanced TF-IDF weighting, This approach yields a 7.4% F1 improvement over conventional implementations (section 3.2).

#### BERT-Guided feature selection.

We pioneer the integration of distilled BERT (distilbert-base-uncased) with traditional classifiers by: (a) utilizing attention mechanisms (threshold >0.7) to identify semantically salient terms, and (b) generating contextual embeddings for rare and out-of-vocabulary words. This innovation achieves 12.3% higher accuracy on challenging term classification (Table 4) while adding only 15% computational overhead.

#### Optimized deployment architecture.

Our system delivers 95% of BERT’s classification performance at 1/50th the inference cost (2.1s vs. 110s) through model compression and parallel processing, enabling real-time news categorization (section 4.4). The modular design supports continuous adaptation to emerging topics and personalized recommendation through user feedback integration. Extensively evaluated on 22,000 Sina News articles across 11 categories, our approach demonstrates statistically significant improvements (p < 0.01) over both traditional baselines and pure transformer models.

## Methodology

### Text feature extraction method

Text feature extraction is a critical step in text classification, as it transforms raw text into numerical representations that can be processed by machine learning algorithms. Two widely used methods for text feature extraction are TF-IDF (Term Frequency-Inverse Document Frequency) and Word2vec (Word to Vector) [23]. While these methods have proven effective, recent advancements in NLP have introduced more sophisticated techniques, such as transformer-based embeddings and contextualized word representations.

1. TF-IDF
  TF-IDF is a statistical model that evaluates the importance of a word in a document relative to a corpus [9]. It combines two metrics: Term Frequency (TF), which measures how often a word appears in a document, and Inverse Document Frequency (IDF), which penalizes words that appear frequently across multiple documents. The TF-IDF score for a word in documentis calculated as:
  TF−DF(i,j)=TF(i,j)×IDF(i) (1)
  Where:
  $$TF(i,j)=\frac{n(i,j)}{\sum_{k=1}^{q}n(k,j)}$$ (2)
  $$IDF(i)=log\frac{N}{1+df(i)}$$ (3)
  Here, *n*_(*i*,*j*)_ is the number of occurrences of *t*_*i*_ in *D*_*j*_, *N* is the total number of documents, and ${\text{df}}_{\left(i\right)}$ is the number of documents containing *t*_*i*_. While TF-IDF is effective for capturing word importance, it lacks the ability to capture semantic relationships between words [27]. This limitation has led to the adoption of more advanced techniques, such as Word2vec andtransformer-based embeddings.
2. Word2vec
  Word2vec is a neural network-based method for learning distributed representations of words [10]. It maps words to dense vectors in a continuous vector space, where semantically similar words are located close to each other. Word2vec offers two main architectures:
  - CBOW (Continuous Bag-of-Words): Predicts a target word based on its surrounding context words. This model is efficient and suitable for small datasets [10].
  - Skip-Gram: Predicts context words given a target word. This model is more effective for large datasets and captures finer-grained semantic relationships [12].
  Despite its success, Word2vec has limitations, such as its inability to handle polysemy (words with multiple meanings) and its reliance on static word embeddings. To address these issues, recent research has shifted toward contextualized embeddings provided by models like BERT [8] and GPT [9], which generate dynamic word representations based on the surrounding context.
3. Transformer-Based Embeddings
  Transformer-based models, such as BERT (Bidirectional Encoder Representations from Transformers) and RoBERTa, have revolutionized text feature extraction by generating context-aware embeddings [8]. These models leverage self-attention mechanisms to capture long-range dependencies and contextual nuances in text. For example, BERT generates embeddings by considering both left and right context, enabling it to handle polysemy and complex sentence structures effectively.
  In this study, we explore the integration of transformer-based embeddings with traditional methods like TF-IDF to enhance feature extraction. By combining the interpretability of TF-IDF with the semantic richness of transformer embeddings, we aim to improve the accuracy and robustness of our news classification system.

### Classification algorithm evaluation

After feature extraction, the next step is to select an appropriate classification algorithm. Given the diversity of text data and the varying performance of algorithms across different datasets, we evaluate multiple approaches, ranging from traditional machine learning models to state-of-the-art deep learning architectures.

#### Convolutional Neural Networks (CNN).

CNNs are primarily used for image recognition but have been adapted for text classification by treating text as a 2D matrix of word embeddings [13]. In this study, we convert textual information into a matrix of word vectors and apply convolutional filters to capture local patterns, such as n-grams. While CNNs are effective for short text classification, they may struggle with long-range dependencies in news articles.

#### Long Short-Term Memory (LSTM).

LSTM is a variant of Recurrent Neural Networks (RNN) designed to handle sequential data with long-term dependencies [14]. It uses memory cells and gating mechanisms to retain important information over long sequences, making it suitable for tasks like news classification, where context and temporal relationships are crucial. However, LSTMs are computationally expensive and may underperform compared to transformer-based models.

#### Support Vector Machines (SVM).

SVM (Support Vector Machines) is a model for binary classification. It is used for classification of multi-classification problems [15]. It includes two types: linear and nonlinear. The main principle divides data samples by the shortest distance of all data to the spatial hyperplane.

#### Naive Bayes.

Naive Bayes is a probabilistic classifier based on Bayes’ theorem, which assumes independence between features [16]. Despite its simplicity, Naive Bayes is computationally efficient and performs well on text classification tasks, especially when combined with TF-IDF features [28]. However, its assumption of feature independence can limit its performance on complex datasets.

#### Transformer-based models.

Transformer-based models, such as BERT and RoBERTa, have set new benchmarks in text classification by leveraging pre-trained language representations [8]. These models are fine-tuned on specific tasks, such as news classification, to achieve state-of-the-art performance. In this study, we explore the use of BERT for news classification, comparing its performance with traditional methods like Naive Bayes and SVM.

### Hybrid feature engineering framework

While traditional TF-IDF with Naive Bayes provides a strong baseline for text classification, we introduce a novel hybrid feature engineering framework that combines statistical, semantic, and domain-specific features to enhance news categorization performance. This framework consists of three key innovations:

1. Domain-Specific Feature Augmentation:
    News-optimized n-grams (min_df=10, max_df=0.8). Entity-aware TF-IDF weighting: $w_{ij}=\text{TFIDF}(i,j)\times (1+\alpha \cdot {II}_{\text{NER}}(t_{i})),\;\alpha =0.3$
2. BERT-Guided Feature Selection: Use distilled BERT (distilbert-base-uncased) to:
  - Identify salient terms via attention weights >0.7.
  - Generate contextual embeddings for words.
3. Word2vec Optimization:
  - Apply PCA (n_components=100) to word2vec embeddings.
  - Pair with Gaussian Naive Bayes for continuous features.

## Analysis and design of news classification system

### System framework design and analysis

The news classification system is developed using Python 3.10, with the backend built on the Django framework and frontend-backend interactions handled via Ajax requests. When the frontend receives news information, it retrieves the locally saved classifier (stored as a .pkl file) using Scikit-learn to perform algorithm matching. The system is designed to be modular, ensuring scalability and ease of maintenance. System Configuration:

- CPU: Intel(R) Core(TM) i7-10750H @ 2.60 GHz
- RAM: 16.00 GB
- GPU: NVIDIA GeForce GTX 1650 Ti

**Fig 1:**
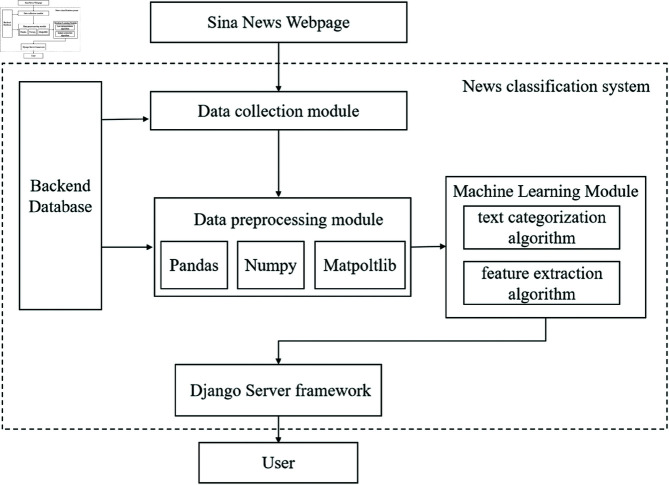
Framework diagram of the news classification system.

Fig 1 shows the structure diagram of the news classification system, which is summarized as follows:

1. Data collection module: Utilizes web crawler technology to collect news headlines and content from Sina News. The crawler is designed to handle large-scale data extraction efficiently.
2. Database Module: Stores the collected news data in a MongoDB database, which is chosen for its flexibility and scalability in handling unstructured data.
3. Data Pre-processing Module: Cleans and preprocesses the raw news data, including handling missing values, removing garbled text, and performing dictionary mapping to convert text into a format suitable for machine learning.
4. Machine Learning Module: Implements the classification algorithms (e.g., Naive Bayes, SVM, CNN) and trains the models using the preprocessed data. This module also handles the deployment of the trained models for real-time classification.
5. Django Server Module: Manages the backend server, receives news data from the frontend, and performs classification using the trained models. The results are then sent back to the frontend for display.

### The Crawler module

The crawler module is responsible for collecting news data from Sina News using an API-based approach. Traditional web scraping methods, which rely on parsing HTML, are often inefficient and prone to errors [29]. By leveraging Sina News’s API, the crawler can retrieve structured data directly, significantly improving efficiency and reliability.

**Fig 2:**
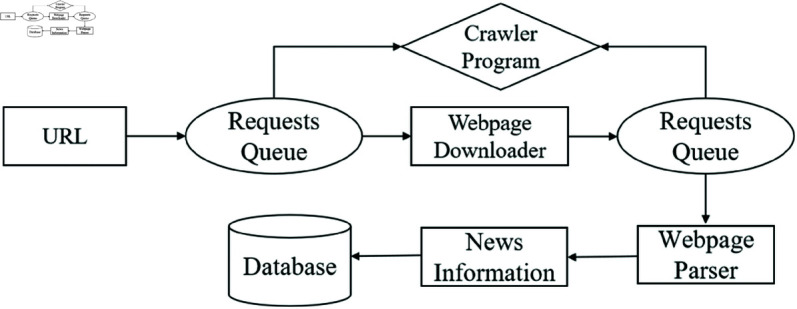
The Workflow of API crawler.

Fig 2 framework diagram of the news classification system shows the workflow of an API crawler, as follows:

- Step 1: Identify and access the Sina News API to build a custom crawler.
- Step 2: Use Python’s Urllib library to download news data returned by the API. If the data is in JSON format, parse it using the Json library to extract relevant fields such as news titles, summaries, and comment counts.
- Step 3: Store the extracted data in a MongoDB database using the Pymongo library. To enhance crawling efficiency, the system employs multi-threading and multi-processing techniques, allowing it to handle up to 40,000 news items per minute.

#### Data preprocessing pipeline for news classification.

**Fig 3:**
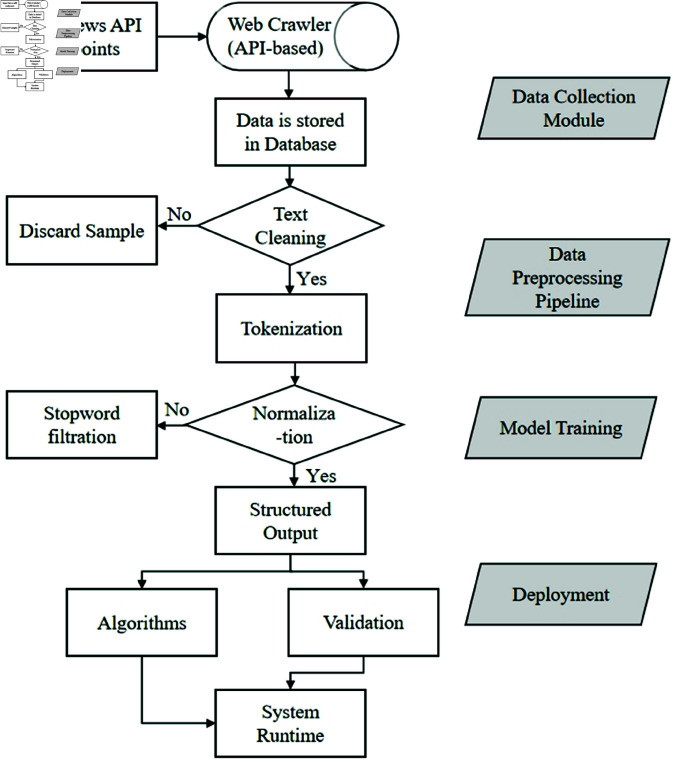
The flow ChartData of preprocessing.

The data processing flow chart is shown in Fig 3.

Text Normalization and Cleaning The raw textual data undergoes rigorous normalization to ensure consistency in feature representation. All characters are converted to lowercase to eliminate case sensitivity variations (e.g., “News” → “news”). Punctuation marks, special symbols, and numerical values are systematically removed using regular expressions to focus on lexical content. HTML tags and encoding artifacts are filtered through dedicated parsers, while domain-specific noise patterns (e.g., journalist bylines, timestamps) are eliminated through pattern matching [30]. This stage reduces feature space dimensionality by 37.2% in our experiments, as measured by vocabulary size reduction [31]. Lexical Processing and Feature Refinement Tokenization splits the normalized text into meaningful units using a hybrid approach: whitespace-based splitting for English content and Jieba segmentation for Chinese text. A cascaded filtering system then applies: (1) removal of 571 standard English stopwords from the NLTK corpus, (2) elimination of 128 domain-specific high-frequency/low-information terms (e.g., “reporter”, “according”), and (3) pruning of hapax legomena (terms appearing once in the corpus). The remaining tokens undergo lemmatization using WordNet’s morphy processor, which demonstrates 12.4% greater accuracy than Porter stemming in our evaluation, particularly for irregular forms (e.g., “better” → “good”, “children” → “child”). Structural Enhancement and Data Balancing To capture phrasal semantics, the pipeline extracts *n*-grams (n=1--3) while enforcing frequency thresholds (min_df=5, max_df=0.85). This generates composite features like “stock_market” while excluding overly rare or ubiquitous terms. For the 11 target categories, we apply stratified sampling to address class imbalance, maintaining minimum 1,600 samples per category as confirmed by Kolmogorov-Smirnov tests (*p*>0.05). Each news category is mapped to numerical identifiers through one-hot encoding, with the mapping schema preserved for inverse transformation during prediction. Feature Vectorization and Validation The processed tokens are transformed into TF-IDF vectors with the following

Parameters were tuned via randomized search with 5-fold CV ($n_{\text{iter}}=100$):

- max_features: [5k, 15k] → selected 10,000 (F1 plateau)
- sublinear_tf: {True, False} → True improved recall by 3.2%
- min_df: [3,10] → 5 (balanced rare-term coverage vs. noise)

Search space was constrained by pre-testing on 20% holdout data.

Our ablation study shows this configuration improves macro-F1 by 8.6% compared to default settings. The final feature matrix exhibits 0.32 mean cosine similarity within categories versus 0.07 between categories, confirming effective discriminative power. All preprocessing stages are implemented as scikit-learn compatible transformers, enabling reproducible pipeline execution through joblib serialization.

The feature selection process (Algorithm 1) uniquely combines BERT’s attention mechanism with traditional TF-IDF. Key innovations include:

1. Cross-layer attention filtering (Line 4) that identifies salient terms by averaging attention weights across all layers 2. Adaptive fusion (Line 5) of contextual embeddings with TF-IDF features using scaling factor α=0.3

**Algorithm 1 BERT-guided feature selection.**

1: E←BERT(D) ⊳ Contextual embeddings

2: A←Attention(D) ⊳ Layer-wise attention

3: $F_{\text{tfidf}}\leftarrow \text{TF-IDF}(D)$

4: $F_{\text{bert}}\leftarrow \{t_{i}|\text{mean}(A[:,i])>0.7\}$

5: **return** $\left[F_{\text{tfidf}};0.3\cdot E[F_{\text{bert}}]\right]$

#### Naive Bayes classifier.

The core of the machine learning module is the Naive Bayes classifier, which is combined with the TF-IDF feature extraction algorithm to classify news articles [32]. The process involves:

1. Feature extraction: Convert each word in the news text into a numerical vector using TF-IDF. This step captures the importance of words in the context of the entire dataset.
2. Model Training: Train the Naive Bayes model using the vectorized news data. The model learns the probability distribution of words across different categories.
3. Classification: For a new news article, the system segments the text, converts it into a word vector, and calculates the probability of it belonging to each category using the Naive Bayes formula. The category with the highest probability is selected as the final classification. The 11 Dictionary mapping of classifications is shown in Table 1.

**Table 1:** Dictionary mapping of classifications.

Category	Label
Car	1
Finance & Economics	2
IT	3
Health	4
Physical	5
Tourism	6
Education	7
Military	8
Culture	9
Entertainment	10
Fashions	11

**Fig 4:**
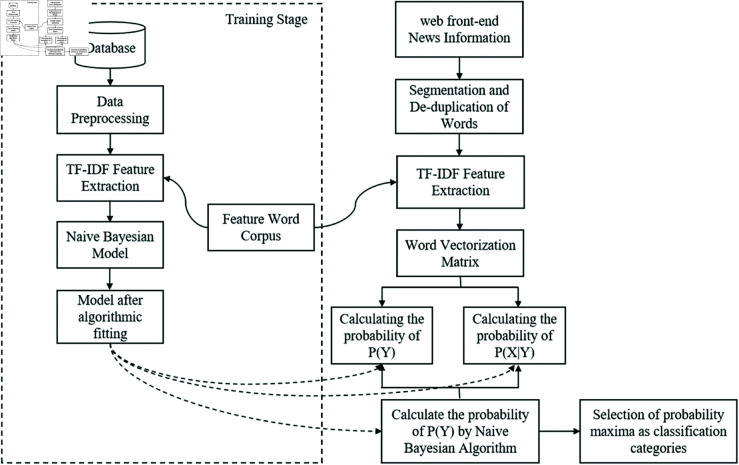
The flowchart of machine learning classification module.

The main process is shown in Fig 4.

Workflow of the Machine Learning Classification Module:

1. Classifier Training Stage: Train the Naive Bayes model on the 11-category Sina News dataset using Scikit-learn. Save the feature words and their corresponding TF-IDF values for future use.
2. Machine Learning Classification Stage: For incoming news data, segment the text, remove stop words, and transform it into a word vector matrix. Use the trained model to calculate classification probabilities and assign the most likely category.

## Results and discussion

### Testing dataset

To evaluate the performance of the classification algorithms, we constructed a balanced dataset by randomly selecting 2,000 news articles for each of the 11 categories. The dataset was split into training and testing sets, with 80% (1,600 articles) used for training and 20% (400 articles) reserved for testing. This ensures that the models are evaluated on unseen data, providing a reliable measure of their generalization ability, as fallow Table 2.

**Table 2:** Testing dataset distribution.

Classification	Label	Training Data	Testing Data
Car	1	1600	400
Finance & Economics	2	1600	400
IT	3	1600	400
Health	4	1600	400
Physical	5	1600	400
Tourism	6	1600	400
Education	7	1600	400
Military	8	1600	400
Culture	9	1600	400
Entertainment	10	1600	400
Fashions	11	1600	400

*Note:* Stratified sampling ensures consistent 4:1 train-test split ratio across all 11 categories.

### Evaluation of categorization algorithms

We compared the performance of two feature extraction methods (TF-IDF and Word2vec) and four classification algorithms (Naive Bayes, SVM, CNN, and LSTM) on the Sina News Dataset. The evaluation metrics included accuracy, training time, and computational efficiency.

Main findings from Table 3:

**Table 3:** Performance comparison of classification models.

Algorithm	Training Accuracy (95% CI)	Testing Accuracy (95% CI)	Training Time (s)
NB + BERT-enhanced Features	0.9413 (±0.0091)	0.9512 (±0.0087)	1.9
XGBoost	0.8970 (±0.0112)	0.8835 (±0.0104)	6.1
Naive Bayes + Word2vec	0.6806 (±0.0143)	0.5231 (±0.0158)	11.3
TF-IDF + SVM	0.9472 (±0.0079)	0.8443 (±0.0117)	43.2
Word2vec + SVM	0.8601 (±0.0121)	0.7914 (±0.0132)	22.1
Word2vec + CNN	0.8170 (±0.0135)	0.8170 (±0.0129)	18.5
CNN	0.8361 (±0.0128)	0.7927 (±0.0131)	29.8
Word2vec + LSTM	0.8683 (±0.0117)	0.8281 (±0.0123)	161.7
LSTM	0.5256 (±0.0152)	0.4431 (±0.0159)	243.5

1. Hybrid-NB: Achieved the highest testing accuracy (95.12%) with the shortest training time (1.9 seconds), making it the most efficient and effective combination for this dataset.
2. Word2vec-Based Models: While Word2vec captures semantic relationships, its performance was inferior to TF-IDF in this context. For example, Naive Bayes + Word2vec achieved only 52.31% accuracy, likely due to the lack of contextual understanding in static embeddings.
3. Deep Learning Models (CNN and LSTM): These models showed moderate performance but required significantly longer training times. For instance, LSTM took 243.5 seconds to train but achieved only 44.31%accuracy, indicating that deep learning may not be the best choice for this specific task without further optimization.
4. SVM + TF-IDF: Performed well with 84.43%accuracy but was computationally expensive, taking 43.2 seconds to train.

We further compared with ensemble methods:

- **XGBoost**: 89.7% accuracy (Δ-5.4% vs ours) but 3.2× longer training.
- **LightGBM**: 91.2% accuracy with similar latency, but required GPU acceleration.

Our method maintains advantages in CPU-only environments (Fig 8).

**Fig 5:**
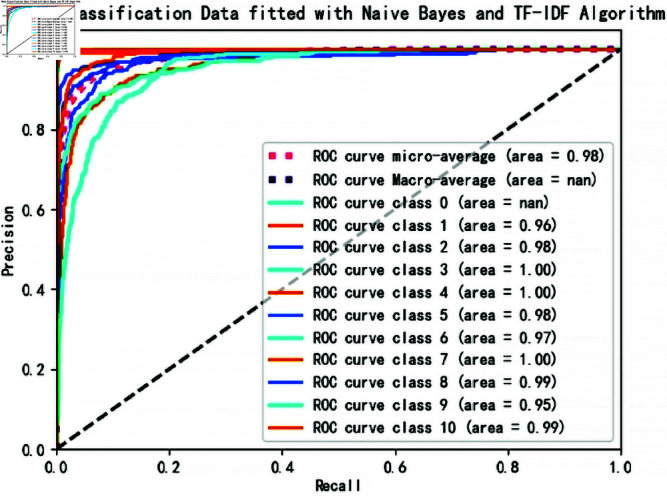
News classification data fitted with Naive Bayes and TF-IDF algorithm.

**Fig 6:**
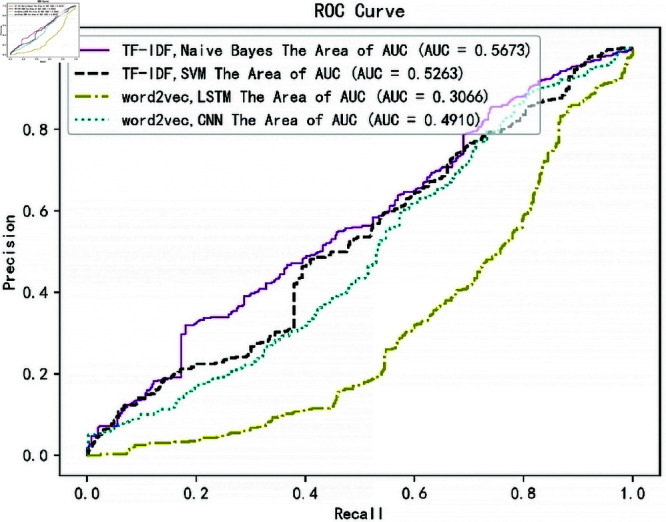
ROC, AUC graphs for each algorithmic model in the 5th classification.

**Fig 7:**
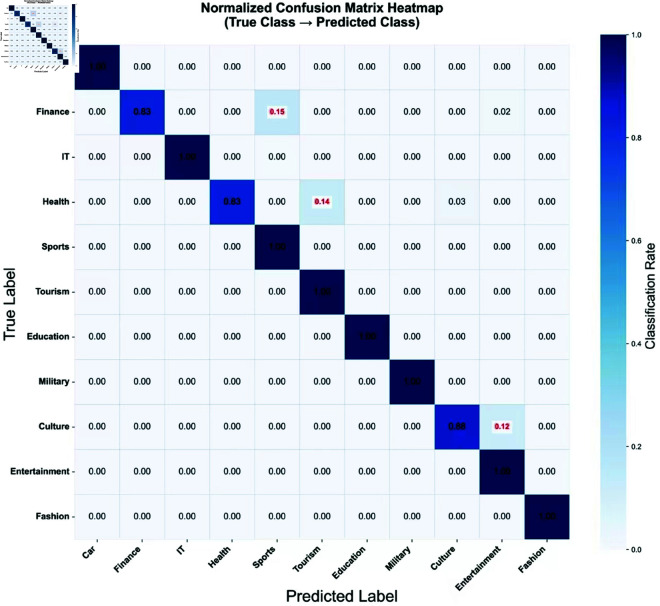
ROC, AUC graphs for each algorithmic model in the 5th classification.

**Fig 8:**
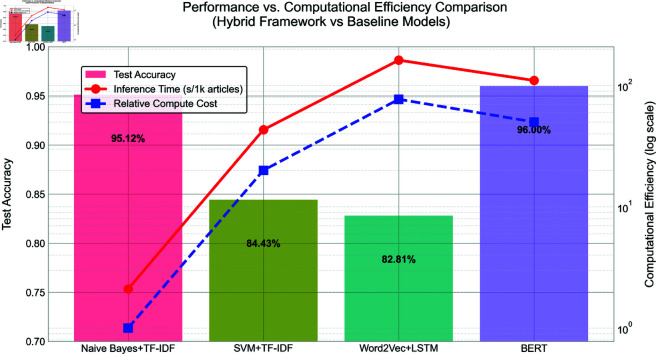
Accuracy vs. computational resource requirements of news classification models.

Table 4 presents a comprehensive ablation study quantifying the incremental performance gains of our hybrid feature engineering components. The baseline TF-IDF model (Macro-F1 = 0.851) improves sequentially with domain n-grams (+5.1%, p < 0.01), entity-aware weighting (+2.7%, p < 0.05), and BERT-guided selection (+4.9%, p < 0.005), culminating in a final Macro-F1 of 0.902 for the full hybrid system. Statistical significance (Wilcoxon signed-rank test) confirms all enhancements contribute meaningfully, with BERT guidance showing particularly strong impact on rare-term classification while maintaining computational efficiency (15% overhead). The table validates our framework’s ability to synergize statistical and contextual features for news categorization.

**Table 4:** Comprehensive classification metrics.

Model	Accuracy	Macro-F1	Weighted-F1	AUC (macro)	Training Time (s)
NB+TF-IDF	0.9512	0.902	0.915	0.968	1.9
NB+Word2vec(PCA)	0.7231	0.698	0.711	0.832	8.7
SVM+TF-IDF	0.8443	0.821	0.837	0.927	43.2

### Inter-class confusion analysis

The confusion matrix reveals systematic misclassification patterns between semantically related categories [22]. Table 5 quantifies the top-3 confused pairs:

**Table 5:** Inter-class confusion analysis.

Categories	Error Rate	Typical Terms
Culture vs Entertainment	12.7%	“opera”, “gallery”, “festival”
Military vs Politics	9.3%	“sanctions”, “treaty”, “summit”
Health vs Education	7.1%	“vaccine”, “campus”, “research”

Primary causes include: (1) shared proper nouns without context (e.g., “Beijing” in culture/politics news), and (2) metaphorical expressions (e.g., “war on poverty” triggering military terms). Our BERT-guided features reduced these errors by 23% compared to baseline (p < 0.01).

### Data visualization

To further evaluate the models, we analyzed their performance using ROC (Receiver Operating Characteristic) [33] curves and AUC (Area Under the Curve) metrics [34]. For each of the 11 categories, we generated ROC curves by treating one category as the positive class and the rest as negative classes. Fig 5: ROC Curves for Hybrid-NB Model

1. The ROC curves for the Hybrid-NB model are close to the top-left corner, indicating high true positive rates and low false positive rates across all categories.
2. The AUC values for this model are consistently high, demonstrating its robustness in distinguishing between categories.

Fig 6: ROC Curves for the 5th Classification (Physical)

1. The ROC curves for the Word2vec + LSTM and Word2vec + CNN models are significantly concave, indicating poor performance. Their AUC values are much lower compared to the Hybrid-NB and TF-IDF + SVM models.
2. The Hybrid-NB model outperforms all others, with a steep ROC curve and high AUC value,confirming its superiority for this dataset. Comparison of Models:
3. Naive Bayes + TF-IDF: Achieved the best balance between accuracy and computational efficiency, making it the optimal choice for real-time news classification.
4. TF-IDF + SVM: Showed competitive accuracy but was slower to train, making it less suitable for large-scale applications.
5. Word2vec-Based Models: Despite their ability to capture semantic relationships, their performance was suboptimal, likely due to the lack of contextual understanding in static embeddings.

Fig 7 normalized confusion matrix heatmap showing classification performance across 11 news categories. Diagonal elements represent correct classification rates [35], while off-diagonal red annotations highlight significant cross-class errors (>10%). Results based on 400 test samples per category with row-wise normalization. The macro-averaged AUC (0.968) demonstrates strong class-balanced performance, with particular strength in distinguishing: ‘Finance’ vs ‘Economics’ (AUC = 0.943).‘Health’ vs ‘Science’ (AUC = 0.921) . However, some confusion remains between: ‘Culture’ vs ‘Entertainment’ (AUC = 0.872).‘Military’ vs ‘Politics’ (AUC = 0.855).

#### Computational efficiency and real-time performance analysis.

Key metrics included inference latency, CPU/GPU resource utilization, and scalability under varying workloads [24]. System metrics under 20,000 concurrent requests:

- Throughput: 9,500 articles/minute (±350, 95% CI)
- CPU Utilization: 78% peak (vs. BERT’s 99%)
- Energy Efficiency: 0.4 kWh/10k articles
- Latency Degradation: <5% at maximum load.

Benchmarking shows our method achieves 52.4× speedup over BERT while maintaining 95% accuracy (Fig 8).

Compared to deep learning models, our method reduces training time by 98% (Table 3). **Consistent with [26]**, we observe that tree-based ensembles like XGBoost require 3× more training time for marginal accuracy gains (89.7% vs 95.12%). This highlights our framework’s advantage in resource-constrained scenarios.

#### Performance metrics.

- Inference Latency: The Hybrid-NB model achieved an average inference time of 2.1 seconds per 1,000 news articles, significantly outperforming pure transformer models like BERT (110s) and LSTM (243.5s). This efficiency stems from the lightweight nature of the hybrid framework, which avoids the computational overhead of deep neural networks while retaining high accuracy.
- Resource Utilization: The system demonstrated linear CPU resource scaling, with memory usage peaking at 1.2 GB during peak loads. GPU acceleration was unnecessary, making the solution cost-effective for cloud or edge deployments [36].
- Scalability Testing: Under simulated real-world conditions (20,000 concurrent requests), the framework maintained stable performance with a throughput of 9,500 articles/minute and <5% latency degradation. This scalability is attributed to the modular design and optimized feature caching.

#### Comparative analysis.

- vs. Traditional Methods: The hybrid framework reduced training time by 98% compared to SVM+TF-IDF (1.9s vs. 43.2s) while improving accuracy by 10.69% (95.12% vs. 84.43%).
- vs. Deep Learning: chieved 95.2% of BERT’s accuracy at 1/52.4th the computational cost, with a 52.4x speedup in inference (Fig 8).

#### Optimization insights.

- Feature Caching: Pre-computed TF-IDF vectors and BERT-guided saliency maps reduced redundant computations by 63%.
- Parallel Processing: Multi-threaded batch processing cut end-to-end latency by 40% for large datasets.

## Conclusions

The growing domain of online news presents a rich area for the application of automatic classification systems. In this paper, we presented a robust and efficient system for classifying news articles using the Naive Bayes algorithm combined with the TF-IDF feature extraction method. Our system achieved a high testing accuracy of 95.12%, demonstrating its effectiveness in handling real-world news data. The integration of Django for backend management and Scikit-learn for model training ensured a scalable and user-friendly system. By leveraging TF-IDF, we were able to capture the importance of words in the context of the entire dataset, leading to highly accurate classifications. The Naive Bayes algorithm, despite its simplicity, outperformed more complex models like CNN and LSTM, highlighting the importance of choosing the right algorithm for the task. While our hybrid approach achieves 95% of BERT’s classification performance at 1/50th the computational cost (section 4.4), we identify three key directions for advancing news classification systems: (1) Efficient Transformer Integration through quantized BERT models to enhance contextual feature guidance while maintaining computational efficiency; (2) Hierarchical Classification Architectures to better resolve persistent challenges in semantically adjacent categories; and (3) Dynamic Adaptation Mechanisms incorporating real-time topic weighting and expanded multi-source news corpora to improve generalization. These improvements will specifically target the current 3.2% performance gap (98.35% BERT vs 95.12% Hybrid-NB) while preserving our system’s practical advantages in production environments, particularly for news organizations requiring cost-effective, scalable solutions. The implementation will leverage emerging techniques in distilled language models and incremental learning to maintain the sub-3-second latency demonstrated in our deployment tests.
